# Cytotoxic lymphocytes in COPD airways: increased NK cells associated with disease, iNKT and NKT-like cells with current smoking

**DOI:** 10.1186/s12931-018-0940-7

**Published:** 2018-12-07

**Authors:** Jonas Eriksson Ström, Jamshid Pourazar, Robert Linder, Anders Blomberg, Anne Lindberg, Anders Bucht, Annelie F. Behndig

**Affiliations:** 1 Department of Public Health and Clinical Medicine, Division of Medicine, Umeå University, 90187 Umeå, Sweden; 2 Swedish Defence Research Agency, Division of CBRN Defence and Security, Umeå, Sweden

**Keywords:** Chronic obstructive pulmonary disease, Disease mechanisms, Lung function decline, Smoking habits, Bronchoalveolar lavage

## Abstract

**Background:**

Cytotoxic lymphocytes are increased in the airways of COPD patients. Whether this increase is driven primarily by the disease or by smoking is not clear, nor whether it correlates with the rate of decline in lung function.

**Methods:**

Bronchoscopy with BAL was performed in 52 subjects recruited from the longitudinal OLIN COPD study according to pre-determined criteria; 12 with COPD and a rapid decline in lung function (loss of FEV_1_ ≥ 60 ml/year), 10 with COPD and a non-rapid decline in lung function (loss of FEV_1_ ≤ 30 ml/year), 15 current and ex-smokers and 15 non-smokers with normal lung function. BAL lymphocyte subsets were determined using flow cytometry.

**Results:**

In BAL fluid, the proportions of NK, iNKT and NKT-like cells all increased with pack-years. Within the COPD group, NK cells – but not iNKT or NKT-like cells – were significantly elevated also in subjects that had quit smoking. In contrast, current smoking was associated with a marked increase in iNKT and NKT-like cells but not in NK cells. Rate of lung function decline did not significantly affect any of the results.

**Conclusions:**

In summary, increased proportions of NK cells in BAL fluid were associated with COPD; iNKT and NKT-like cells with current smoking but not with COPD. Interestingly, NK cell percentages did not normalize in COPD subjects that had quit smoking, indicating that these cells might play a role in the continued disease progression seen in COPD even after smoking cessation.

**Trial registration:**

Clinicaltrials.gov identifier NCT02729220.

**Electronic supplementary material:**

The online version of this article (10.1186/s12931-018-0940-7) contains supplementary material, which is available to authorized users.

## Background

In COPD patients, the airway lumen is infiltrated by T cells and increased numbers of neutrophils and macrophages [1, 2]. The latter are thought to be the orchestrators of the inflammation, releasing chemokines that attract T cells and other lymphocytes, monocytes and neutrophils as well as mediating the release of proteases such as MMP-9 [3].

Among T cells, cytotoxic CD8^+^ cell types predominate [4]. The reason for this is not fully understood, but bacterial colonization and viral infections have been suggested to activate the cytotoxic response [5]. The proportions of natural killer (NK) [6], natural killer T (NKT)-like [6] and invariant natural killer [7] (iNKT; sometimes also referred to as NKT Type I or Classical NKT cells [8]) are also increased in the airways of COPD patients, but the role of these cells in the pathogenesis of the disease and whether this increase is in fact due to COPD or to current smoking is not clear [9].

Taken together, the above described immunological changes are thought to be signs of a state of chronic inflammation, which over time leads to structural transformation of the airways, airway obstruction and respiratory symptoms [10]. However, the pace at which these changes occur – i.e. the rate of disease progression– varies greatly between individuals [11]. A rapid annual decline in lung function (LF) has been recognized as a clinical phenotype of COPD [12] and is associated with a poor prognosis [11]. Smoking cessation is known to reduce the rate of decline [12], but beyond that the reason why some patients experience a more rapid and some a slower disease progression is not well understood.

In this study, the aim was to assess the distribution of cytotoxic lymphocytes in COPD in general and their association with disease status, smoking status and a rapid/non-rapid decline in LF in particular.

## Methods

### Subjects

52 subjects participated in this cross-sectional study; 12 with COPD and a rapid decline in LF, 10 with COPD and a non-rapid decline in LF, 15 current and ex-smokers with normal LF (Ever-smokers) and 15 non-smokers with normal LF (Non-smokers).

All subjects were recruited, according to pre-determined criteria, from the longitudinal OLIN COPD study [13], providing spirometry data over time. The recruitment process has been described in a previous report on the ‘Respiratory and Cardiovascular Effects in COPD’ (KOLIN) project [14].

COPD was defined using the Global Initiative for Obstructive Lung Disease (GOLD) spirometric criterion [15]; all COPD subjects had GOLD spirometric grades 2–3 at time of inclusion. Rapid decline was defined as a loss of forced expiratory volume in one second (FEV_1_) ≥ 60 ml/year and non-rapid decline as a loss ≤30 ml/year; both measured over a period of at least five years and calculated using data from the OLIN COPD study as (FEV_1_ at recruitment – FEV_1_ at follow-up)/number of years of follow-up [14]. COPD and Ever-smokers groups consisted of both current and ex-smokers, all with a smoking history of at least 10 pack-years. Ex-smokers had stopped smoking since at least 12 months. Subjects with medical conditions contradicting bronchoscopy and/or inflammatory conditions or medication expected to affect the outcome of the study were excluded from participation [14]. Subject demographics and basic characteristics are given in Table 1.

**Table 1:** Basic characteristics of the study population, by spirometry classification and smoking status

Part 1: Characterizing the inflammation				
	All COPD subjects (COPD) *n* = 22	Ever-smokers with normal LF (EvS) *n* = 15	Non-smokers with normal LF (NoS) *n* = 15	*p*
Female:Male	6:16	8:7	4:11	
Age^a^	65 ± 7	67 ± 6	66 ± 8	NS
BMI^b^	26 ± 3	26 ± 2	28 ± 5	NS
Current:Ex-smokers^b^	11:11	3:12	0:0	
Pack-years^b^	36 ± 14	18 ± 9	0	**p** **= 0.0002** COPD vs EvS; **p < 0.0001** COPD vs NoS
FEV_1_, percent of predicted^a^	61.5 ± 17	108 ± 19	103 ± 17	**p** **< 0.0001** COPD vs EvS; **p < 0.0001** COPD vs NoS;
FEV_1_/VC ^a^	0.53 ± 0.11	0.73 ± 0.04	0.78 ± 0.04	**p < 0.0001** COPD vs EvS; **p < 0.0001** COPD vs NoS;
BAL-recovery, %^c^	42 ± 17	61 ± 12	63 ± 10	**p** **= 0.0006** COPD vs EvS: **p** **= 0.0001** COPD vs NoS
Annual decline in FEV_1_, ml^a^	57 ± 42	NA	NA	
Rapid:Non-rapid decline^b^	12:10	NA	NA	
Part 2: Separating the effect of smoking from that of COPD				
	COPD current smokers (CCuS) *n* = 11	COPD ex-smokers (CExS) *n* = 11	Ex-smokers with normal LF (ExS) *n* = 12	*p*
Female:Male^a^	2:9	4:7	6:6	
Age^a^	61 ± 5	69 ± 6	67 ± 7	**p** **= 0.0048** CCuS vs CExS
BMI^b^	25 ± 4	26 ± 2	26 ± 2	NS
Pack-years^b^	38 ± 9.3	33 ± 18	18 ± 9	**p** **= 0.0444** CCuS vs CExS; **p** **= 0.0158** CExS vs ExS
FEV_1_, percent of predicted^a^	61 ± 16	62 ± 18	107 ± 17	**p < 0.0001** CExS vs ExS
FEV_1_/VC ^a^	0.53 ± 0.12	0.53 ± 0.12	0.73 ± 0.04	**p < 0.0001** CExS vs Exs
BAL-recovery, %^c^	47 ± 17	37 ± 17	64 ± 7	**p < 0.0001** CExS vs ExS
Annual decline in FEV_1_, ml^a^	73 ± 44	39 ± 36	NA	**p** **= 0.0199** CCuS vs CExS
Rapid:Non-rapid decline^b^	8:3	4:7	NA	
Part 3: COPD and a rapid/non-rapid of decline in lung function				
	COPD rapid decline in LF *n* = 12	COPD non-rapid decline in LF *n* = 10	*p*	
Female:Male	2:10	4:6		
Age^a^	63 ± 7	67 ± 6	**p** **= 0.0358**	
BMI^b^	26 ± 3	25 ± 3	NS	
Current:Ex-smokers^b^	8:4	3:7		
Pack-years^b^	37.5 ± 16	33 ± 11	NS	
FEV_1_, percent of predicted^a^	60 ± 15	63 ± 19	NS	
FEV_1_/VC ^a^	0.52 ± 0.12	0.54 ± 0.11	NS	
BAL-recovery, %^c^	44 ± 16	40 ± 19	NS	
Annual decline in FEV_1_, ml^a^	86 ± 29	16 ± 16	**p < 0.0001**	

Values given as mean ± SD unless indicated differently. Statistical comparisons between the three groups were made using Kruskal Wallis test and a *p*-value < 0.05 was considered significant. If the Kruskal Wallis test indicated significance, the Mann-Whitney U-test was used for post hoc analysis. *NS:* Not significant. *Pack-years*: (number of cigarettes smoked per day/20) × number of years smoked. *FEV*_*1*_: Forced Expiratory Volume in one second. *VC*: Vital Capacity. *Annual Decline in FEV*_*1*_*, ml*: calculated using data from the OLIN COPD study as (FEV_1_ at recruitment – FEV_1_ at follow-up)/number of years of follow-up, based on highest value pre- or post-bronchodilation

^a^At inclusion in the current study

^b^At identification in the OLIN COPD study

^c^At time of bronchoscopy in the current study

### Study design

**Fig. 1:**
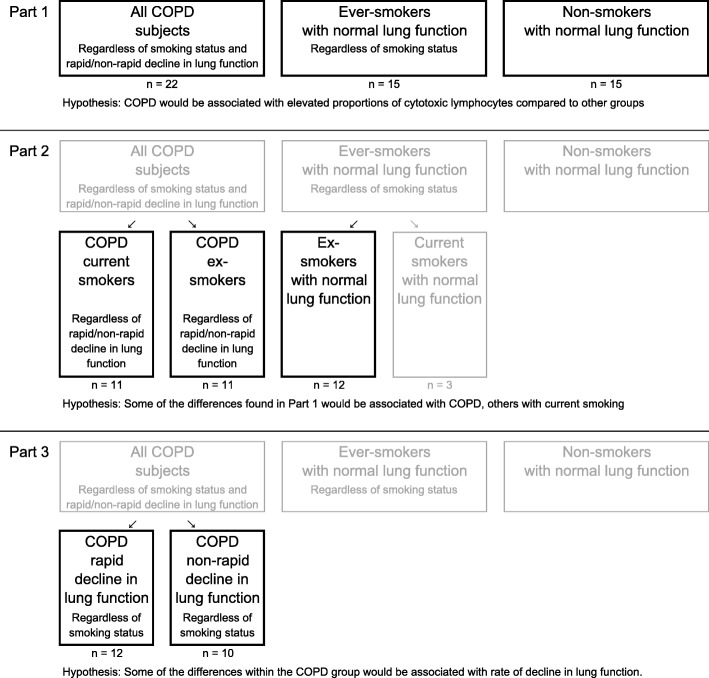
Study design

The current study was divided into three parts, each testing a separate hypothesis. Groups were either merged or divided into subgroups depending on the hypothesis tested (Fig. 1).

In part 1 the inflammatory characteristics and distribution of cytotoxic lymphocytes were examined using as big groups as possible. It was hypothesized that COPD would be associated with elevated proportions of cytotoxic lymphocytes and therefore all COPD subjects (current and ex-smokers) were compared to to Ever-smokers and Non-smokers.

In part 2 the hypothesis was that some of the differences found in part 1 would be associated with current smoking and others with COPD. To separate between these, we compared 1) COPD current smokers to COPD ex-smokers; differences between these groups would likely be related to smoking status (since disease status is the same for both groups); and 2) COPD ex-smokers to ex-smokers with normal LF; differences between these groups would likely be related to disease status (since smoking status is the same for both groups).

In part 3 COPD subjects were split into two groups according to rate of decline in LF to test the hypothesis that some of the differences found in part 1 would be associated with a rapid decline.

### Spirometry

Dynamic spirometry variables were measured using a dry volume spirometer, Mijnhardt Vicatest 5, the Netherlands, following the American Thoracic Society/European Respiratory Society guidelines [16]. Vital Capacity (VC) was defined as the highest value of forced and slow vital capacity. If FEV_1_ was lower than 80% of predicted (using Swedish spirometric reference values [17]) or if FEV_1_/VC was below 0.70, reversibility testing was performed. The highest value out of pre- and post-bronchodilatation FEV_1_ and VC was used in the analysis.

### Bronchoscopy

All bronchoscopies were performed by one medical team but at two different locations – the Division of Respiratory Medicine and Allergy, Department of Medicine, Sunderby Central Hospital of Norrbotten, Luleå, Sweden and the Division of of Respiratory Medicine and Allergy, Department of Medicine, University Hospital, Umeå, Sweden. Topical anesthesia was achieved using lidocaine. Subjects were premediated with 1.0 mg of atropine given subcutaneously 30 min before the procedure, some also received midazolam 4–8 mg per os. A flexible video bronchoscope was inserted through the mouth via a mouthpiece with the subject in the supine position. Bronchoalveolar lavage (BAL) was performed by infusing three aliquots of 60 ml of sterile sodium chloride (0.9%), pH 7.3 at 37 °C in the middle lobe or lingula, the fluid was gently sucked back after each infusion and pooled into a tube placed in iced water. The recovered BAL fluid (BALF) was immediately transported to the laboratory for analysis. Bronchial wash (2 × 20 ml) and biopsies were also performed but not included in the analysis in the current study.

BAL could not be performed on three COPD subjects due to problems tolerating the bronchoscopy procedure. In one COPD subject, BALF recovery was too low to perform flow cytometry analysis, although differential cell count of leukocytes was possible. No exacerbations were reported in the four weeks prior to bronchoscopy.

### Flow cytometry analysis

Cell staining and data acquisition were performed at one centralized location. BALF lymphocyte subsets were determined using a FACSCalibur™ (Becton Dickinson) flow cytometer. BALF cells were centrifuged and adjusted to a final concentration of 10^6^ cells/ml. For each test, different antibody panels conjugated with either phycoerytrin-Cy5 (PE Cy5), fluorescein isothiocyanate (FITC), phycoerytrin (PE), Allophycocyanin (APC), Peridinin Chlorophyll Protein Complex (PerCP) or Peridinin Chlorophyll Protein Complex-Cy5.5 (PerCPCy5.5) were combined. Appropriate isotype-matched controls were used in all experiments. The suppliers for the antibodies against TCR Vβ11 and TCR Vα24 were Beckman Coulter (Brea, CA, USA) and eBiodcience, Inc. (Thermo Fisher Scientific, Sweden) respectively, whereas the remaining antibodies used in this investigation were purchased from Becton Dickinson (San Jose, CA, USA). Each test tube contained 200–400 μl of cell suspension (10^6^ cells/ml) to which 10 μl of each antibody was added. After the staining procedure [18], analysis was performed using FACSCalibur™. The lymphocyte population was gated based on the cells’ physical characteristics in a region according to their characteristic forward scatter (FCS) and side scatter (SSC) profiles. 6000–9000 cells were collected in CD3^+^ gate per test tube and percentage of CD3 subpopulation was counted out of gated CD3^+^ lymphocytes and furthermore out of gated subpopulations (Table 2). To ensure that autofluorescence did not influence the results, we stained for CD45/CD14 and compared that to the results of lymphocyte and macrophage gating. Flow cytometry data were acquired and analysed using CellQuest Software (Becton Dickinson).

**Table 2:** Lymphocyte populations and FACS staining characteristics

Population	Subpopulation	Staining characteristics	When given in percent, calculated as proportion of
T cells	–	CD3^+^	–
T helper cells	–	CD3^+^ CD4^+^	CD3^+^
Cytotoxic T cells	–	CD3^+^ CD8^+^	CD3^+^
NK cells	–	CD3^−^ CD16^+^ CD56^+^	Cells with typical lymphocyte size and intracellular granulation
iNKT cells	**–**	CD3^+^ (TCR)αβ^+^ Vα24^+^ Vβ11^+^	CD3^+^ (TCR)αβ^+^
NKT-like cells	–	CD3^+^ CD16^+^ CD56^+^	CD3^+^
NKT-like cells	CD4^+^ NKT-like cells	CD3^+^ CD4^+^ CD16^+^ CD56^+^	CD3^+^
NKT-like cells	CD8^+^ NKT-like cells	CD3^+^ CD8^+^ CD16^+^ CD56^+^	CD3^+^

### Statistical analysis

To analyze the data, a two-step approach was used. In the first step, the investigated cell populations and subpopulations were analyzed using group-wise comparisons. For statistical comparisons between more than two groups, the Kruskal-Wallis test was used and a *p*-value < 0.05 was considered significant. If the Kruskal-Wallis test indicated significance, the Mann-Whitney U-test was carried out for post-hoc comparison between two groups. In the second step, cytotoxic cell populations with significant between-group differences were examined further using multivariable mixed effects regression models. These models were performed by specifying the response variable as the number of cells in the population evaluated (numerator) and the remaining number of lymphocytes (denominator) and incorporating subjects as random effect (random intercepts) in the linear predictor of a generalized linear model with a binomial error distribution. In the mixed effects regression models, adjustments were evaluated for age, sex and smoking (where applicable). Smoking was evaluated both as a categorical variable (smoking status) and as a continuous variable (pack-years). Statistical analysis was performed using IBM SPSS Statistics (version 23) and, for calculating mixed effects regression models, statistical software package R (version 3.3.3; R Development Core Team, R foundation for Statistical Computing, Vienna, Austria).

## Results

### Part 1 – Airway inflammation in COPD

**Fig. 2:**
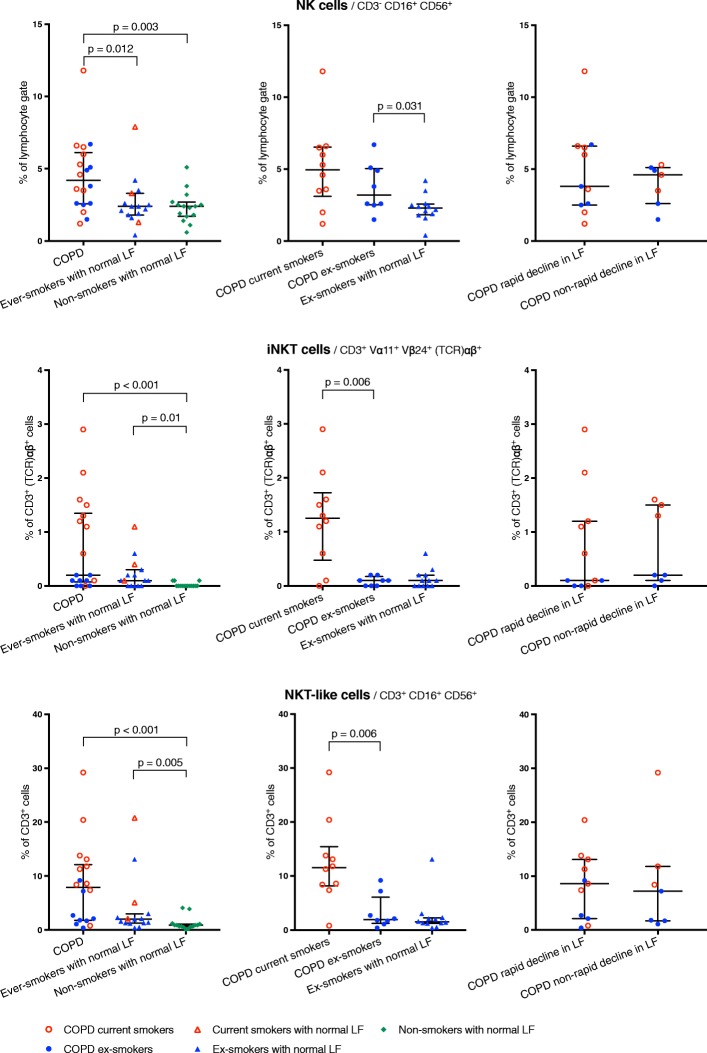
Part 1, 2 and 3: NK, iNKT and NKT-like cell populations in BAL fluid. Data shown as median and IQR. *LF*: lung function. Shown *p*-values calculated using the Mann-Whitney U-test. See Additional file 1 for corresponding data in tables

The proportion of NK cells was higher in the COPD group compared to both Ever-smokers and Non-smokers. In iNKT and NKT-like cells, no significant difference was found comparing COPD to Ever-smokers, but both these groups had increased proportions compared to Non-smokers (Fig. 2). NKT-like cell subpopulations exhibited the same pattern as the NKT-like population as a whole (Additional file 1: Table S3). The differential cell count showed no significant differences (Additional file 1: Table S1a).

### Part 2 – Separating the effect of smoking from that of COPD

**Fig. 3:**
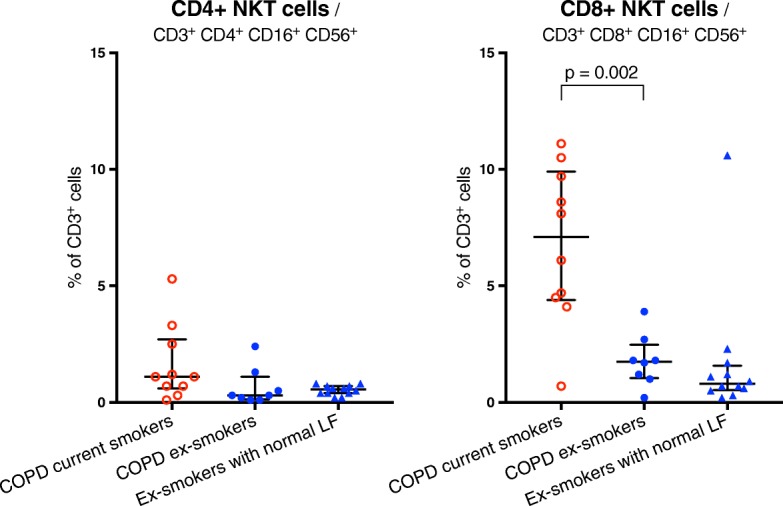
Part 2: NKT-like cell subpopulations in BAL fluid. Data shown as median and IQR. *LF*: lung function. Shown p-values calculated using the Mann-Whitney U-test. See Additional file 1 for corresponding data in tables

In NK cells, no significant difference was found between COPD current and COPD ex-smokers. However, both these groups had higher proportions of NK cells than ex-smokers with normal LF. Airway iNKT and NKT-like cells were significantly increased in COPD current compared to COPD ex-smokers, but there was no significant difference between COPD ex-smokers and ex-smokers with normal LF (Fig. 2). Among NKT-like cells, the CD8+ but not the CD4+ subpopulation was significantly increased in COPD current compared to COPD ex-smokers (Fig. 3). The proportion of cytotoxic T-cells did not differ significantly between groups (Additional file 1: Table S2).

The mixed effects-regression analysis showed a statistically significant relationship between pack-years and increased proportions of NK, iNKT and NKT-like cells (OR (95% CI): 1.02 (1.01–1.02), 1.11 (1.06–1.17) and 1.04 (1.02–1.06) respectively; *p* < 0.0001 for all; among all subjects). Increased NK cells were also significantly associated with COPD (OR (95% CI) 1.50 (1.05–2.13); *p* = 0.018; among ex-smokers, adjusted for pack-years).

The models also showed that the relative increase due to current smoking in the proportion of cytotoxic cells was largest for iNKT cells and smallest for NK cells (current smokers vs. ex-smokers; OR (95% CI): iNKT cells 1007 (163–7119), NKT-like cells 9.14 (4.64–17.98); and NK cells 2.16 (1.39–3.35)).

The differential cell count of leukocytes showed increased numbers of macrophages in COPD current compared to COPD ex-smokers (*p* = 0.003). No other significant differences in differential cell counts were found (Additional file 1: Table S1a).

### Part 3 – COPD and a rapid/non-rapid of decline in lung function

Between-group comparisons of the differential cell count of leukocytes, cell populations and subpopulations showed no significant differences (see Additional file 1: Tables S1, S2 and S3). The lack of association between rapid/non-rapid decline in LF and NK, iNKT and NKT-like cells was confirmed in the mixed effects-regression models, which included adjustments for age and smoking status.

## Discussion

We have demonstrated increased proportions of NK cells in BALF from COPD patients compared to both Ever-smokers and Non-smokers. This increase endured even after smoking cessation, as ex-smokers with COPD had significantly higher proportions of NK cells than ex-smokers with normal LF (Fig. 2).

These results seemingly contradict those of the two previous studies which indicated that NK cell proportions in BALF depend on current smoking status rather than COPD [19, 20]. However, neither of those studies included ex-smokers with normal LF as a control group and could thus not compare ex-smokers with and without COPD in order to identify enduring disease-specific alterations.

In the current study, the mixed model regression analysis showed that increased NK cells are associated both with cumulative smoking (pack-years) *and* with COPD. The latter association remained significant after adjustment for pack-years.

Our results echo those of a previous study of mice models, in which NK cells were identified as a candidate persistence determinant of chronic airway inflammation following cigarette smoke exposure [21]. BALB/c mice were exposed to 6 cigarettes/day, 6 days/week for 16 weeks leading to an inflammatory profile in BALF similar to that of human COPD patients. After 12 weeks of non-exposure NK cells were still higher in the exposed group compared to controls.

Furthermore, in vitro experiments on cells from human lung tissue have shown that NK cells from COPD patients are more prone to kill autologous lung epithelial cells than NK cells from non-obstructive subjects [22, 23]. This spontaneous cytotoxicity also increased with worsening FEV_1_% predicted, supporting a potential role of NK cells in emphysema progression [22]. Increased expression of NK cell activating receptor ligands (such as MICA and MICB) by lung epithelial cells [22] as well as differences in the NK cells themselves have been proposed to explain these findings [23].

Taken together, these results indicate that NK cells may play an important role in the continued disease progression seen in COPD patients even after smoking cessation.

iNKT and NKT-like cells exhibited a pattern distinctly different from that of NK cells. In these cell populations, the main between-group difference was found not between COPD and non-COPD subjects, but rather between smokers and non-smokers.

Part 1 of the study showed increased iNKT and NKT-like cells in the two groups that consisted of smokers (COPD and Ever-smokers) compared to Non-smokers. In part 2, COPD current smokers had significantly higher proportions than COPD ex-smokers while no differences were seen between ex-smokers with COPD and ex-smokers with normal LF (Fig. 2). This pattern was then verified in the mixed effects-regression analysis where no statistical relationship between COPD and iNKT or NKT-like cells was found, but instead showed that these cells were heavily influenced by current smoking.

The definition of iNKT cells is not consistent throughout the literature. In this study, the term iNKT cells is used for the CD1d-dependent, α-GalCer reactive, Vα24^+^ Vβ11^+^ population which sometimes is also referred to as Type I NKT cells or Classical NKT cells [8]. Currently, the best way to identify iNKT in FACS is by using α-GalCer-loaded CD1D tetramers. In the current study CD3^+^ (TCR)αβ^+^ Vα24^+^ Vβ11^+^ was used to define this cell type. While these markers combined are fairly specific for iNKT cells, we cannot rule out that some other (non-invariant) T cells also expressed these markers and thus were included in the cell counts.

Functionally, iNKT cells are thought to play an important role in the pathogenesis of COPD. In a study on C57BL/6 mouse models, Pichavant et al. showed that exposure to cigarette smoke (5 cigarettes/day, 5 days/week, for up to 12 weeks) led to the accumulation of activated iNKT cells in the lungs [24]. In another study, BALB/c mice received weekly intranasal administrations of α-GalCer in order to induce iNKT cell activation. After eight weeks, these mice models had developed pulmonary emphysema as well as molecular and inflammatory features similar to those of COPD [25].

Pichavant et al. also showed that smoking-related oxidative damage may be mediated through iNKT cells [24]. In a model of acute oxidative stress, wild type mice exposed to cumen hydroperoxide (CHP) – a compound that triggers lipid peroxidation – exhibited increased airway resistance and recruitment of neutrophils into the lungs. In knock-out mice lacking iNKT cells no change in LF or airway inflammation was seen following CHP exposure.

In humans, one previous study reported increased levels of iNKT cells in lung tissue from COPD compared to non-COPD subjects [7]. The results of the current study extend those findings by providing evidence of increased iNKT cells also in BALF and, more importantly, that such an increase may be associated more closely with current smoking than with COPD (Fig. 3).

NKT-like cells are not well-studied in the context of COPD. The results of two previous studies suggest that increased proportions of these cells are related to current smoking and not to COPD [19, 20]. This was confirmed in the current study which additionally showed that increased NKT-like cells are associated not only with current but also with *cumulative* smoking (pack-years).

In the current study, NKT-like cells were defined as CD3^+^ CD16^+^ CD56^+^ cells. While this is a commonly used gating strategy for this population, it should be noted that it is likely to include cells not associated with the NKT cell lineage such as (TCR)γδ^+^ T cells. Also, the antibodies used in the analysis of NKT-like cell subpopulations do not cover all known variants and the results thereof should thus be interpreted with caution. However, it could be noted that the smoking-related increase seen in NKT-like cells as a whole seems to be driven mainly by CD8^+^ NKT-like cells which predominantly produce Th1-type cytokines [26], while CD4^+^ NKT-like cells, capable of producing both Th1- and Th2-type cytokines [26], were not significantly increased by smoking (Fig. 3). These changes could contribute to tipping the Th1-Th2 balance in the lungs of smokers in a more pro-inflammatory direction. More functional studies of NKT-like cells are needed in order to better understand their role in COPD.

Contrary to our hypothesis, no significant differences in cytotoxic cell populations were found between COPD subjects with a rapid and a non-rapid decline in LF. One possible explanation could be that these clinically distinct phenotypes differ immunologically in aspects other than relative numbers of cytotoxic cells; e.g. immune cell activation, level of cytotoxicity and/or levels of regulatory immune cells. While beyond the scope of this study, these aspects should be investigated in further research on COPD rapid/non-rapid decliners. Another explanation could be that the current study is simply underpowered to detect such differences. There are very few previous studies on immunological changes related to rate of decline in LF and there is no established definition of rapid/non-rapid decline in the literature, making power calculations highly uncertain.

In the current study, data on lymphocyte populations and subpopulations are presented in relative and not absolute cell numbers. The reason for this is that BAL recovery volumes, as in previous studies [19, 27], were found to be lower in COPD subjects compared to both non-smokers and ex-smokers with normal lung function [14]. We therefore believe that relative cell numbers better reflect differences in the inflammatory response when comparing these groups.

One strength of this study is the inclusion of not only Non-smokers, but also ex-smokers with normal LF acting as control groups. This enables comparisons of groups, between which only one major characteristic differ (e.g. ex-smokers with and without COPD), allowing us to better pinpoint the driving mechanism behind the reported immunological differences. Another strength is the use of a multivariable mixed effects-regression model to validate that between-group differences are indeed associated with the major difference in characteristic and not with other factors such as pack-years or age.

A limitation to this study is that women were underrepresented in the COPD rapid decline and Non-smokers groups (Table 1). Thus, we could not evaluate sex-specific differences, nor rule out that results were affected by these differences in group composition. There are interventional studies demonstrating sex-specific differences in the inflammatory response to tobacco smoke [28] as well as cross-sectional studies indicating that that women are more susceptible to tobacco smoke [29]. We have previously reported that the participation of COPD subjects in bronchoscopy studies is negatively affected by the large burden of co-morbidities within that group. As a result, the recruitment process might be disabled even if the basis for recruitment is large [14]. Nevertheless, to allow analyses stratified for sex, future studies should consider study population structure and size.

Another limitation is that the current study did not include a sufficient number of current smokers with normal LF. Because of this, comparisons between current smokers with and without COPD were not possible. And while disease specific changes are likely better detected comparing ex-smokers with normal LF to ex-smokers with COPD (current smoking is such a strong factor that it may mask changes related to the disease), it would have been a strength if such changes found could have been verified in a comparison between current smokers with and without COPD.

## Conclusions

NK, iNKT and NKT-like cell proportions in BALF all increased with pack-years. Increased NK cells were also associated with COPD, while increased iNKT and NKT-like cells were associated with current smoking but not with COPD. Interestingly, NK cell percentages did not normalize in COPD subjects that had quit smoking, suggesting that these cells might play a role in the continued disease progression seen in COPD even after smoking cessation. Contrary to our hypothesis, no significant differences were found between COPD subjects with a rapid and a non-rapid decline in LF. Further research is needed to understand the underlying processes resulting in these two phenotypes.

## Additional file

Additional file 1: **Table S1a.** Differential cell counts of leukocytes of in BAL fluid, given in number of cells/ml*10^4^. **Table S1b.** Differential cell counts of leukocytes of in BAL fluid, given in percent. **Table S2.** Flow cytometry analysis of lymphocytes in BAL fluid, given in percent. **Table S3.** Flow cytometry analysis of NKT-like cell subpopulations in BAL fluid, given in percent. (DOCX 28 kb)

## Abbreviations

BAL: Bronchoalveolar lavage
BALF: Bronchoalveolar lavage fluid
CI: Confidence interval
COPD: Chronic obstructive pulmonary disease
Ever-smokers: Ever-smokers with normal lung function
FACS: Fluorescence-activated cell sorting
FEV_1_: Forced expiratory volume in one second
FVC: Forced vital capacity
GOLD: Global initiative for chronic obstructive lung disease
LF: Lung function
MMP9: Matrix metalloproteinase 9
NK cells: Natural killer cells
NKT-like cells: Natural killer T cell
Non-smokers: Non-smokers with normal lung function
OLIN COPD study: Obstructive Lung Disease in Northern Sweden Chronic Obstructive Pulmonary Disease study
OR: Odds ratio
VC: Vital capacity
α-GalCer: α-galactosylceramide
