# Supplementary material for: Cytotoxic lymphocytes in COPD airways: increased NK cells associated with disease, iNKT and NKT-like cells with current smoking
Source: Respir Res. 2018 Dec 7;19:244. doi: 10.1186/s12931-018-0940-7 (PMC6286566; doi:10.1186/s12931-018-0940-7)
Supplement: Supplementary file 1 — Table S1a. Differential cell counts of leukocytes of in BAL fluid, given in number of cells/ml*104. Table S1b. Differential cell counts of leukocytes of in BAL fluid, given in percent. Table S2. Flow cytometry analysis of lymphocytes in BAL fluid, given in percent. Table S3. Flow cytometry analysis of NKT-like cell subpopulations in BAL fluid, given in percent. (DOCX 28 kb) [file 12931_2018_940_MOESM1_ESM.docx]

Additional file 1

**Table 1a Differential cell counts of leukocytes of in BAL fluid, given in number of cells/ml*10^4^**

| **Part 1: Characterizing the inflammation** | | | | |
| --- | --- | --- | --- | --- |
|  | **COPD**  **n = 19** | **Ever-smokers with normal LF**  **n = 15** | **Non-smokers with normal LF**  **n = 15** | **p** |
| Macrophages | 17 (11-27) | 14 (9.3-31) | 11 (8.6-16) | NS |
| Neutrophils | 0.18 (0.088-0.81) | 0.11 (0.049-0.23) | 0.1 (0.044-0.17) | NS |
| Lymphocytes | 1.8(0.78-2.6) | 1.6(1.3-3.6) | 2.1(1.4-3.8) | NS |
| Eosinophils | 0.077 (0-0.37) | 0.022 (0-0.2) | 0.027 (0-0.044) | NS |
| Mast cells | 0.029 (0-0.11) | 0.0043 (0-0.049) | 0.017 (0.0056-0.02) | NS |

Data are given as median with IQR. Statistical comparisons between the three groups were made using Kruskal Wallis test and a p-value < 0.05 was considered significant. *NS*: Not significant.

| **Part 2: Separating the effect of smoking from that of COPD** | | | | |
| --- | --- | --- | --- | --- |
|  | **COPD**  **current smokers**  **(CCuS)**  **n = 10** | **COPD**  **ex-smokers**  **(CExS)**  **n = 9** | **Ex-smokers with normal LF (ExS)**  **n = 12** | **p** |
| Macrophages | 22 (19-34) | 11 (8.4-15) | 13 (8.7-17) | **p = 0.003**  CCuS vs CExS |
| Neutrophils | 0.17 (0.081-0.72) | 0.18 (0.065-1.5) | 0.11 (0.053-0.22) | NS |
| Lymphocytes | 1.8 (0.92-2.7) | 1.8 (0.75-2.6) | 1.9 (0.83-3.4) | NS |
| Eosinophils | 0.11 (0-0.24) | 0.068 (0.013-0.44) | 0.02 (0-0.032) | NS |
| Mast cells | 0.093 (0.022-0.13) | 0.0051 (0-0.029) | 0.0014 (0-0.059) | NS |

Data are given as median with IQR. Statistical comparisons between the three groups were made using Kruskal Wallis test and a p-value < 0.05 was considered significant. If the Kruskal Wallis test indicated significance, the Mann-Whitney U-test was used for post hoc analysis for comparison of CExS vs CCuS and CExS vs ExS. A p-value < 0.05 was considered significant. *NS*: Not significant.

| **Part 3: COPD and a rapid/non-rapid decline in lung function** | | | |
| --- | --- | --- | --- |
|  | **COPD**  **rapid decline  in lung function**  **n = 11** | **COPD**  **non-rapid decline  in lung function**  **n = 8** | **p** |
| Macrophages | 21 (13-27) | 13 (8.3-26) | NS |
| Neutrophils | 0.18 (0.094-0.81) | 0.13 (0.038-0.88) | NS |
| Lymphocytes | 1.9 (1-3.2) | 1.6 (0.71-2.1) | NS |
| Eosinophils | 0.068 (0-0.13) | 0.12 (0.0063-0.52) | NS |
| Mast cells | 0.065 (0.022-0.11) | 0.0049 (0-0.095) | NS |

Data are given as median with IQR. Statistical comparisons between the two groups were made using the Mann-Whitney U-test and a p-value < 0.05 was considered significant. *NS*: Not significant.

**Table 1b Differential cell counts of leukocytes of in BAL fluid, given in percent**

| **Part 1: Characterizing the inflammation** | | | | |
| --- | --- | --- | --- | --- |
|  | **COPD**  **n = 19** | **Ever-smokers with normal LF (EvS)**  **n = 15** | **Non-smokers with normal LF (NoS)**  **n = 15** | **p** |
| Macrophages | 88 (86-93) | 86 (80-93) | 83 (76-87) | NS |
| Neutrophils | 1.0 (0.40-2.0) | 0.60 (0.20-1.2) | 0.80 (0.40-1.0) | NS |
| Lymphocytes | 7.6 (5.2-13.2) | 12 (4.6-19.2) | 15 (12-23) | **p = 0.009** COPD vs NoS |
| Eosinophils | 0.40 (0-1.0) | 0.20 (0-0.40) | 0.20 (0-0.40) | NS |
| Mast cells | 0.15 (0-0.45) | 0.025  (0-0.15) | 0.10 (0.050-0.15) | NS |

Data are given as median with IQR. Statistical comparisons between the three groups were made using Kruskal Wallis test and a p-value < 0.05 was considered significant. If the Kruskal Wallis test indicated significance, the Mann-Whitney U-test was used for post hoc analysis for comparison of COPD vs EvS, COPD vs NoS and EvS vs NoS. A p-value < 0.05 was considered significant. *NS*: Not significant.

| **Part 2: Separating the effect of smoking from that of COPD** | | | | |
| --- | --- | --- | --- | --- |
|  | **COPD**  **current smokers**  **(CCuS)**  **n = 10** | **COPD**  **ex-smokers**  **(CExS)**  **n = 9** | **Ex-smokers with normal LF (ExS)**  **n = 12** | **p** |
| Macrophages | 93 (88-94) | 87 (65-90) | 85 (78-92) | **p = 0.047**  COPD vs NoS |
| Neutrophils | 0.80 (0.35-1.9) | 1.4 (0.50-4.9) | 0.80 (0.25-0.1.4) | NS |
| Lymphocytes | 5.5 (3.6-12) | 9.0 (7.6-17) | 14 (7.8-20) | NS |
| Eosinophils | 0.30 (0-0.70) | 0.40 (0.10-1.9) | 0.20 (0-0.20) | NS |
| Mast cells | 0.28 (0.094-0.52) | 0.075 (0-0.21) | 0.013 (0-0.43) | NS |

Data are given as median with IQR. Statistical comparisons between the three groups were made using Kruskal Wallis test and a p-value < 0.05 was considered significant. If the Kruskal Wallis test indicated significance, the Mann-Whitney U-test was used for post hoc analysis for comparison of CExS vs CCuS and CExS vs ExS. A p-value < 0.05 was considered significant. *NS*: Not significant.

| **Part 3: COPD and a rapid/non-rapid decline in lung function** | | | |
| --- | --- | --- | --- |
|  | **COPD**  **rapid decline  in lung function**  **n = 11** | **COPD**  **non-rapid decline  in lung function**  **n = 8** | **p** |
| Macrophages | 91 (86-93) | 88 (81-93) | NS |
| Neutrophils | 1.0 (0.40-2.4) | 0.80 (0.25-1.7) | NS |
| Lymphocytes | 6.0 (5.2-13) | 8.3 (5.1-11) | NS |
| Eosinophils | 0.20 (0-0.60) | 0.50 (0.10-2.2) | NS |
| Mast cells | 0.28 (0.13-0.50) | 0.063 (0-0.18) | NS |

Data are given as median with IQR. Statistical comparisons between the two groups were made using the Mann-Whitney U-test and a p-value < 0.05 was considered significant. *NS*: Not significant.

**Table 2 Flow cytometry analysis of lymphocytes in BAL fluid, given in percent**

| **Part 1: Characterizing the inflammation** | | | | |
| --- | --- | --- | --- | --- |
|  | **COPD**  **n = 18** | **Ever-smokers with normal LF**  **(EvS)**  **n = 15** | **Non-smokers with normal LF**  **(NoS)**  **n = 15** | **p** |
| T helper cells | 66 (47-78) | 80 (57-87) | 77 (72-85) | NS |
| Cytotoxic T cells | 29 (18-46) | 17 (10-36) | 20 (13-24) | NS |
| NK cells | 4.2 (2.6-6.1) | 2.4 (1.8-3.3) | 2.4 (1.7-2.7) | **p = 0.003**  COPD vs NoS;  **p = 0.012**  COPD vs EvS |
| iNKT cells | 0.2 (0.075-1.4) | 0.1 (0-0.3) | 0 (0-0) | **p < 0.001**  COPD vs NoS;  **p = 0.01**  EvS vs NoS |
| NKT-like cells | 7.9 (1.8-12) | 2.0 (1.3-3.0) | 0.9 (0.6-1.1) | **p < 0.001** COPD vs NoS;  **p = 0.005**  EvS vs NoS |

Data are given as median with IQR. Percentage calculated out of gated cells, see main article Table 2. Statistical comparisons between the three groups were made using Kruskal Wallis test and a p-value < 0.05 was considered significant. If the Kruskal Wallis test indicated significance, the Mann-Whitney U-test was used for post hoc analysis for comparison of COPD vs EvS, COPD vs NoS and EvS vs NoS. A p-value < 0.05 was considered significant. *NS*: Not significant.

| **Part 2: Separating the effect of smoking from that of COPD** | | | | |
| --- | --- | --- | --- | --- |
|  | **COPD**  **current smokers**  **(CCuS)**  **n = 10** | **COPD**  **ex-smokers**  **(CExS)**  **n = 8** | **Ex-smokers with normal LF (ExS)**  **n = 12** | **p** |
| T helper cells | 57 (38-78) | 71 (55-82) | 81 (73-88) | NS |
| Cytotoxic T cells | 38 (19-54) | 23 (15-42) | 15 (8.4-22) | NS |
| NK cells | 5.0 (3.1-6.5) | 3.2 (2.5-5.1) | 2.3 (1.8-2.6) | **p = 0.031**  CExS vs ExS |
| iNKT cells | 1.3 (0.48-1.7) | 0.1 (0-0.18) | 0.1 (0-0.2) | **p = 0.006**  CCuS vs CExS |
| NKT-like cells | 12 (8.2-15) | 2.0 (1.3-6.1) | 1.5 (1.2-2.3) | **p = 0.006**  CCuS vs CExS |

Data are given as median with IQR. Percentage calculated out of gated cells, see main article Table 2. Statistical comparisons between the three groups were made using Kruskal Wallis test and a p-value < 0.05 was considered significant. If the Kruskal Wallis test indicated significance, the Mann-Whitney U-test was used for post hoc analysis for comparison of CExS vs CCuS and CExS vs ExS. A p-value < 0.05 was considered significant. *NS*: Not significant.

| **Part 3: COPD and a rapid/non-rapid decline in lung function** | | | |
| --- | --- | --- | --- |
|  | **COPD**  **rapid decline**  **in lung function**  **n = 11** | **COPD**  **non-rapid decline**  **in lung function**  **n = 7** | **p** |
| T helper cells | 63 (47-78) | 73 (46-74) | NS |
| Cytotoxic T cells | 32 (18-50) | 25 (19-44) | NS |
| NK cells | 3.8 (2.5-6.6) | 4.6 (2.6-5.1) | NS |
| iNKT cells | 0.1 (0-1.2) | 0.2 (0.1-1.5) | NS |
| NKT-like cells | 8.6 (2.1-13) | 7.2 (1.7-12) | NS |

Data are given as median with IQR. Percentage calculated out of gated cells, see main article Table 2. Statistical comparisons between the two groups were made using the Mann-Whitney U-test. A p-value of < 0.05 was considered significant. *NS*: Not significant.

**Table 3 Flow cytometry analysis of NKT-like cell subpopulations in BAL fluid, given in percent**

| **Part 1: Characterizing the inflammation** | | | | |
| --- | --- | --- | --- | --- |
|  | **COPD**  **n = 18** | **Ever-smokers with normal LF**  **(EvS)**  **n = 15** | **Non-smokers with normal LF**  **(NoS)**  **n = 15** | **p** |
| CD4^+^ NKT-like cells | 0.7 (0.28-1.6) | 0.6 (0.4-0.8) | 0.2 (0.1-0.3) | **p = 0.003** COPD vs NoS;  **p < 0.001**  EvS vs NoS |
| CD8^+^ NKT-like cells | 4 (1.6-8.2) | 1.1 (0.6-2.3) | 0.4 (0.2-0.6) | **p < 0.001** COPD vs NoS;  **p = 0.005**  EvS vs NoS |

Data are given as median with IQR. Percentage calculated out of gated cells, see main article Table 2. Statistical comparisons between the three groups were made using Kruskal Wallis test and a p-value < 0.05 was considered significant. If the Kruskal Wallis test indicated significance, the Mann-Whitney U-test was used for post hoc analysis for comparison of COPD vs EvS, COPD vs NoS and EvS vs NoS. A p-value < 0.05 was considered significant. *NS*: Not significant.

| **Part 2: Separating the effect of smoking from that of COPD** | | | | |
| --- | --- | --- | --- | --- |
|  | **COPD**  **current smokers**  **(CCuS)**  **n = 10** | **COPD**  **ex-smokers**  **(CExS)**  **n = 8** | **Ex-smokers with normal LF (ExS)**  **n = 12** | **p** |
| CD4^+^ NKT-like cells | 1.1 (0.6-2.7) | 0.3 (0.13-1.1) | 0.55 (0.4-0.7) | NS |
| CD8^+^ NKT-like cells | 7.1 (4.4-9.9) | 1.8 (1.1-2.5) | 0.8 (0.53-1.6) | **p = 0.002**  CCuS vs CExS |

Data are given as median with IQR. Percentage calculated out of gated cells, see main article Table 2. Statistical comparisons between the three groups were made using Kruskal Wallis test and a p-value < 0.05 was considered significant. If the Kruskal Wallis test indicated significance, the Mann-Whitney U-test was used for post hoc analysis for comparison of CExS vs CCuS and CExS vs ExS. A p-value < 0.05 was considered significant. *NS*: Not significant.

| **Part 3: COPD and a rapid/non-rapid decline in lung function** | | | |
| --- | --- | --- | --- |
|  | **COPD  rapid decline**  **in lung function**  **n = 11** | **COPD**  **non-rapid decline**  **in lung function**  **n = 7** | **p** |
| CD4^+^ NKT-like cells | 0.7 (0.3-1.2) | 0.7 (0.1-2.5) | NS |
| CD8^+^ NKT-like cells | 4.1 (1.8-8.1) | 3.9 (1.2-8.6) | NS |

Data are given as median with IQR. Percentage calculated out of gated cells, see main article Table 2. Statistical comparisons between the two groups were made using the Mann-Whitney U-test. A p-value of < 0.05 was considered significant. *NS*: Not significant.
